# Optogenetic Activation of Astrocytes Reduces Blood-Brain Barrier Disruption *via* IL-10 In Stroke

**DOI:** 10.14336/AD.2023.0226

**Published:** 2023-10-01

**Authors:** Qian Suo, Lidong Deng, Tingting Chen, Shengju Wu, Lin Qi, Ze Liu, Tingting He, Heng-Li Tian, Wanlu Li, Yaohui Tang, Guo-Yuan Yang, Zhijun Zhang

**Affiliations:** ^1^Shanghai Jiao Tong Affiliated Sixth People’s Hospital, and School of Biomedical Engineering, Shanghai Jiao Tong University, Shanghai, China.; ^2^Department of Neurosurgery, Shanghai Jiao Tong University Affiliated Sixth People's Hospital, Shanghai Jiao Tong University, Shanghai, China.; ^3^Department of Neurology, Shanghai Tenth People's Hospital, Tongji University, Shanghai, China

**Keywords:** astrocyte, blood-brain barrier, interleukin-10, optogenetic, stroke

## Abstract

Optogenetics has been used to regulate astrocyte activity and modulate neuronal function after brain injury. Activated astrocytes regulate blood-brain barrier functions and are thereby involved in brain repair. However, the effect and molecular mechanism of optogenetic-activated astrocytes on the change in barrier function in ischemic stroke remain obscure. In this study, adult male GFAP-ChR2-EYFP transgenic Sprague-Dawley rats were stimulated by optogenetics at 24, 36, 48, and 60 h after photothrombotic stroke to activate ipsilateral cortical astrocytes. The effects of activated astrocytes on barrier integrity and the underlying mechanisms were explored using immunostaining, western blotting, RT-qPCR, and shRNA interference. Neurobehavioral tests were performed to evaluate therapeutic efficacy. The results demonstrated that IgG leakage, gap formation of tight junction proteins, and matrix metallopeptidase 2 expression were reduced after optogenetic activation of astrocytes (*p*<0.05). Moreover, photo-stimulation of astrocytes protected neurons against apoptosis and improved neurobehavioral outcomes in stroke rats compared to controls (*p*<0.05). Notably, interleukin-10 expression in optogenetic-activated astrocytes significantly increased after ischemic stroke in rats. Inhibition of interleukin-10 in astrocytes compromised the protective effects of optogenetic-activated astrocytes (*p*<0.05). We found for the first time that interleukin-10 derived from optogenetic-activated astrocytes protected blood-brain barrier integrity by decreasing the activity of matrix metallopeptidase 2 and attenuated neuronal apoptosis, which provided a novel therapeutic approach and target in the acute stage of ischemic stroke.

## INTRODUCTION

Astrocytes can passively support neuronal development and survival, or actively regulate synaptic transmission and blood-brain barrier (BBB) integrity [1]. Astrocyte activation is a critical feature of an ischemic stroke. Activated astrocytes play a detrimental role by releasing inflammatory factors, such as IL-6, TNF-α, IL-1α, IL-1β, interferon γ (IFNγ), and free radicals [2]. It can also exhibit protective effects after brain injury by releasing interleukin-10 (IL-10), IL-33, VEGF, GDNF, and so on [2-4]. However, the control of their production and diverse functions remains unknown. Activated astrocyte ablation markedly increases neuronal death and exacerbates tissue degeneration after central nervous system (CNS) injury [5]. Therefore, it is very important to understand this specific functional transformation of astrocytes in response to ischemic stroke.

Optogenetics is a unique technique for manipulating cell activity with high specificity and temporal precision [6]. Channelrhodopsin 2 (ChR2)-expressing astrocytes can be controlled by blue laser exposure and have become a useful tool for understanding the roles of astrocyte activation in neural communication for regulating brain functions [7, 8]. Increasing evidence indicates that optogenetically manipulated astrocytes can regulate brain function via gliotransmitters [9-11]. Optogenetic manipulation of astrocytes provides direct evidence of the active role of astrocytes at the circuit level [12]. Photo-stimulation of ChR2 expressed astrocytes increases calcium influx, which induces the release of cytokines and gliotransmitters to regulate the activity of adjacent neurons [13]. Astrocytes cover and interact closely with microvessels through the endfeet to regulate BBB function [14]. BBB integrity is crucial for restoring blood flow and reestablishing a microenvironment for neural repair [15, 16]. This prompted us to explore the effect of activated astrocytes on the BBB after ischemic stroke.

IL-10 is an anti-inflammatory cytokine with immunoregulatory effects in the brain [17, 18]. The expression of IL-10 increases after brain injury, which promotes neuronal and glial cell survival and inhibits the inflammatory response [18-20]. In the CNS, astrocytes and microglia are the potential sources of IL-10 [21, 22]. IL-10 secretion from astrocytes induced by cerebral ischemia could exert an anti-apoptotic effect on injured neurons via the TLR2/NF-κB signaling pathway, which may improve learning and memory dysfunction after ischemic injury [21]. We hypothesized that optogenetic activation of astrocytic calcium-influx via ChR2 could release IL-10 to improve BBB integrity and subsequently reduce neuronal death during the acute phase of ischemic stroke.

## MATERIALS AND METHODS

### Animal experimental design

Animal experiments in this study were performed following the ARRIVE guidelines and animal protocols were approved by the Institutional Animal Care and Use Committee (IACUC) of Shanghai Jiao Tong University, Shanghai, China. All procedures were performed to minimize the pain or discomfort in the animals. In our study, we used transgenic rats GFAP-ChR2-EYFP (Sprague-Dawley background) produced by the Institute of Neuroscience, Chinese Academy of Sciences [23]. Seventy-two rats were randomly divided into six groups in the experiment when they were 8-10 weeks old and weigh 250-300 grams, including:1) Sham/NS = sham group (N = 6); 2) Sham/S = sham with laser stimulation group (N = 6); 3) PT/NS = photothrombotic without laser stimulation group (N = 15); 4) PT/S = photothrombotic with laser stimulation group (N = 15); 5) PT/S/sh (scramble) = photothrombotic with laser stimulation and sh (scramble) adeno-associated virus (AAV) group (N = 15); 6) PT/S/sh (IL-10) = photothrombotic with laser stimulation and sh (IL-10) AAV group (N = 15).

### Photothrombotic (PT) ischemic model

Experimental animals were anesthetized with 1.5%-2% isoflurane in the mixed gas of 30% O_2_ /68.5%-68% NO through a mask and placed in a stereotactic frame. Cut vertically in the middle of the eye to the neck, peel off the scalp and periosteum, and expose the skull. A round skull drill was used to thin a circle positioned on the skull, centered 3 mm left lateral to the bregma, with 1.5 mm as the diameter, and covered other parts to restrict the illuminated area using black tape that carries a hole with a diameter of 3 mm. Photoillumination with a green laser (532 nm Green diode pumped solid state (DPSS) Laser, 80 mW, Shanghai Laser & Optics Century Co., Ltd. (SLOC), GL532T3-100FC) was implemented for 15 min after Rose Bengal (0.05 mg/g, Sigma-Aldrich, 33000-5G) was injected through the tail vein. Using a pointed skull drill to get a hole near the infarction area of sensorimotor cortex to insert a ceramic fiber (200 μm optical fiber, both sides are penetrated, the length of the exit section is 2.5 mm, Ximu Instrument Co., Ltd., Shanghai, China) that could be used for laser stimulation. After the operation, three skull nails (cross thread, 3 mm in length and 1.0 mm in diameter) and denture base-resin (Zhangjiang Biomaterials Co., Ltd, China) were used to fix the fiber near the peri-infarct area.

### Laser stimulation

In the laser stimulation group, astrocytes of GFAP-ChR2-EYFP transgenic rats were activated by a 473 nm pulsed blue laser (473 nm Blue DPSS Laser, 20 Hz, 0.55 mW, Shanghai Laser & Optics Century Co., Ltd. (SLOC), BL473T3-050FC). We performed laser stimulation at 24, 36, 48, and 60 h after PT, lasting 15 min each, which has been proven to be effective [23].

### Viral vector production and injection

The AAV was packaged commercially (HanBio, Shanghai, China). After purification, the virus titer was determined by real-time PCR (1.4×10^12^ V. g/ml). The short hairpin RNA (shRNA) sequence complementary to the site on IL-10 mRNA was designed using the siDESIGN center (Dharmacon, Horizon Discovery Group Co) on a computer, which has been verified and used by researchers [24]. We chose GFAP as the promoter of miR-30-based shRNA cassettes, and the red fluorescent label of mCherry was connected, a plasmid which can target and interfere with the synthesis of IL-10 by astrocytes was synthesized, and it was named HBAAV2/9-GFAP-mir30-r-IL-10-mCherry.

The interference sequence of IL-10 was designed as follows. Forward: 5′-GCTGAAGACCCTCTGGATA CA-3′, Reverse: 5′-TGTATCCAGAGGGTCTTCAGC-3′. Three weeks before the establishment of the photothrombotic model, we selected two sites around the infarcted area, and injected 1 μl virus (2 μl Hamilton needle, 0.1 μl/min) with stereotactic positioning. The injection sites were 4 mm to the left of the bregma, 1.5 mm forward, and 2 mm to the left of the bregma, 1.5 mm backward (Supplementary Fig. 1A and 1B). After the injection, stay for 10 min to ensure success.

### Neurobehavioral function examination

Neurological tests were conducted with the modified neurologic severity score (mNSS) [25]. It is a comprehensive score for evaluating the degree of neurological impairment, taking into account the motor, reflex and sensory abilities of experimental animals. On days 1 and 3 in the PT rats, another investigator who was blinded to the experimental groups applied mNSS with a range of 0-14 points on the rats. A score of 10 to 14 indicated severe injury, 5 to 9 indicated moderate injury, and 1 to 4 indicated mild injury. The normal and maximum injury scores are 0 and 14, respectively.

The ladder rung walking test can be used to assess skilled walking and interlimb coordination in rats. The walking process of PT rats was recorded using a camera on days 1 and 3. Using the 7-category scale evaluation of the position of the front paw on the rungs according to previously described methods, a lower score represents a more serious injury. The analysis was performed by calculating the fault scores [26].

### Primary astrocyte culture

Primary astrocytes were isolated from the GFAP-ChR2-EYFP transgenic rats. Briefly, the cerebral cortex was gently dissected from the brains of newborn rats and washed three times with Dulbecco's modified Eagle’s medium (DMEM, HyClone, Logan, UT, USA). The cerebral cortices were trypsinized (using 0.25% (vol/vol) trypsin) at 37 °C for 7 min, suspended in FBS (Gibco, Carlsbad, NM), and filtered through a 70 μm filter (Millipore, Burlington, MA). The dissociated cortical cells were plated on 6-well plates coated with poly-D-lysine (PDL, Sigma-Aldrich) and cell slides at a density of 7×10^5^ cells/well and then treated with DMEM containing 10% (vol/vol) FBS (Gibco, Carlsbad, NM) and 1% (vol/vol) penicillin streptomycin antibiotics (HyClone, Logan, UT). Mixed glial cells were cultured in a humidified incubator at 37 °C with 5% CO_2_ for 7-10 days, and the culture medium was replaced every 3 days.

### Calcium imaging recording

A Rhod-3 Calcium Imaging Kit (R10145, Thermo Fisher) was used to detect activation of GFAP-ChR2-EYFP transgenic rat cortical astrocytes using a pulsed blue laser (473 nm). The medium was removed from primary astrocytes and the cells were washed twice with phosphate-buffered saline (PBS, Meilunbio, Shanghai, China). Then loading buffer (20 µL 100X PowerLoad™ concentrate; 2 µl 10 mM Rhod-3 AM; 20 µl 250 mM Probenecid; 1958 µl PBS) was used to incubate the cells in the dark for 30 min at room temperature and washed cells three times with PBS. The cells were imaged under a confocal microscope (TCS SP5, Leica, Germany) under illumination with a blue laser.

### Brain infarct and edema volume measurements

Adult rats in all groups were sacrificed on day 3 after PT treatment and were transcardially perfused with PBS. The brains were quickly frozen in isopentane (78-78-4, Macklin, China) at -80 °C for 1 min and then directly frozen at -80 °C for one day. Twenty μm thick frozen coronary brain sections were cut with a microtome and attached to the slide from the start to the end of the infarction. The volume of cerebral infarction and edema was measured using cresyl violet staining. The infarct volume was the total volume of the contralateral hemisphere minus the volume of the normal area of the ipsilateral hemisphere. The edema volume is the total volume of the ipsilateral side minus the volume of the contralateral hemisphere. The infarct or edema volume was calculated using the following formula: V = ∑h/3*[∆S_n_ +(∆S_n_ *∆S_n+1_ )^1/2^+∆S_n+1_ ], where ΔS_n_ and ΔS_n +1_ represent the infarct or edema areas of two adjacent sections, and h represents the distance between two adjacent sections (*h* = 300 μm) [27].

### IgG staining for the evaluation of BBB integrity

Frozen sections were moved to room temperature, fixed with 4% paraformaldehyde (PFA, Sinopharm Chemical Reagent, China) for 5 min, the membrane was broken with 0.3% Triton X-100 solution for 10 min, and blocked with 0.3% H_2_ O_2_ in methanol for 30 min. The ABC reagent kit (Cat#SP-0022, Bioss, Beijing, China) was used for IgG staining, DAB substrate kit (Cat#SK-4100, Vector, San Francisco, CA) for color development, and hematoxylin for counterstaining for 5 min. Gradient dehydration and neutral resin mounts were then performed. Four fields near the cortical infarction were selected under a microscope (Leica) to analyze the integrated optical density (IOD) with image j (NIH, Bethesda, MD, USA).

### Immunofluorescence staining

For immunofluorescence staining of tight junction proteins, brain sections were fixed with ice-cold methanol at -20 °C for 10 min and incubated in 0.3% Triton X-100 solution for 30 s. For fluorescence staining of other antibodies, the brain sections were fixed with 4% PFA for 10 min and 0.3% Triton X-100 solution for 10 min. All sections were blocked with 1% bovine serum albumin (BSA, GBICO, MA) for 1 hour, and then incubated with rabbit-anti ZO-1 (1:200; Cat#61-7300, Invitrogen, Carlsbad, CA), mouse-anti Occludin (1:200; Cat#33-1500, Invitrogen), goat-anti CD31 (1:200; Cat#AF3628, R&D, Minneapolis, MN), mouse-anti IL10 (1:100; Cat#sc-365858, Santa Cruz, CA), mouse-anti IL-10R (1:100; Cat#sc-365374, Santa Cruz), rabbit-anti NeuN (1:200; Cat#abn78, Millipore, Burlington, MA), rabbit-anti MAP2 (1:200, 8707, Cell Signaling Technology, MA, USA), goat-anti GFAP (1:200; Cat#ab53554, Abcam), rabbit-anti CD11b (1:200, ab75476, Abcam), rabbit-anti MMP2 (1:200; Cat#ab92536, Abcam), rabbit-anti MMP9 (1:200; Cat#ab76003, Abcam), rabbit-anti caspase-3 (1:200; Cat#9662, Cell Signaling Technology), and rabbit-anti cleaved caspase-3 (1:200; Cat#AF7022, Affinity) overnight at 4 °C under humidified conditions, respectively. After washing with PBS, the sections were incubated with the corresponding fluorescent-conjugated secondary antibodies for 1 h at 37 °C and observed under a confocal microscope (TCS SP5; Leica). All the fluorescent-conjugated secondary antibodies were listed as follows: Alexa Fluor 594-conjugated donkey anti-mouse secondary antibody (1:400; Cat#A21203, Invitrogen), Alexa Fluor 594-conjugated donkey anti-rabbit secondary antibody (1:400; Cat#A21207, Invitrogen), Alexa Fluor 647-conjugated donkey anti-mouse secondary antibody (1:400; Cat#A31571, Invitrogen), Alexa Fluor 647-conjugated donkey anti-rabbit secondary antibody (1:400; Cat#A31573, Invitrogen), Alexa Fluor 647-conjugated donkey anti-goat secondary antibody (1:400; Cat#A21447, Invitrogen), Alexa Fluor 405-conjugated donkey anti-mouse secondary antibody (1:400; Cat#ab175658, Abcam), and Alexa Fluor 405-conjugated donkey anti-goat secondary antibody (1:400; Cat#ab175664, Abcam). The genuine target staining was distinguished from the background with a secondary antibody only.

Statistical analysis of the immunofluorescence staining data is shown in Supplementary Fig. 2. The average value of the four rectangular areas is the brain slice value, and each mouse counts four brain slices, and the average value of the four brain slices is the rat value.

### TUNEL staining

The TUNEL fluorescence method was performed using the One Step TUNEL Apoptosis Assay Kit (Cat#MA0224, Meilunbio, Shanghai, China) to detect the apoptosis in cortical neurons. The operating procedure was the same as that of the immunofluorescence treatment, but it takes 0.3% Triton X-100 was used to break the membrane for 30 min, and TdT enzyme was mixed with fluorescent labeling solution at a ratio of 1:9 to be used as the diluent of secondary antibody.

The TUNEL chromogenic method used a TUNEL cell apoptosis detection kit (Cat#C1091, Beyotime, Shanghai, China) combined with immunofluorescence staining to observe neuronal apoptosis in the bright field. Frozen sections were fixed with 4% PFA for 30 min and washed three times with PBS for 10 min each time. The membrane was then broken with 0.3% Triton X-100 for 30 min, and incubated with a 0.3% hydrogen peroxide solution prepared in PBS for 20 min. After washing three times, the solution labeled with biotin was used as the primary antibody dilution of NeuN and incubated at 37 °C for 90 min in the dark. After incubation with the labeled reaction termination solution at room temperature for 10 min, streptavidin-HRP working solution was used as the dilution of the immunofluorescence secondary antibody, incubated at 37 °C for 60 min in the dark, and then observed after DAB staining. Data were analyzed using Prism GraphPad 9 (GraphPad Software, San Diego, CA, USA).

### Western blot analysis

Western blot analysis was performed as described in previous research [28]. The ipsilateral cortex was dissolved in extraction buffer during cryosectioning. Equal amounts of protein (30 μg) were loaded onto 7.5%, 10%, and 12.5% SDS -polyacrylamide gel electrophoresis (Epizyme, Shanghai, China), and transferred onto polyvinylidene fluoride (PVDF) membrane (0.2 or 0.45 µm, RIPA, Millipore, Burlington, MA). After blocking for 15 min with protein-free rapid blocking buffer (Epizyme, Shanghai, China), the membrane was incubated with the primary antibodies (1:1,000) at 4 °C for 16 h in the dark, washed with 1×TBST buffer (Epizyme, Shanghai, China), and incubated with horseradish peroxidase-conjugated secondary antibody (anti-mouse or anti-rabbit IgG, 1:5000, Invitrogen) at room temperature for 1 h. Immunoblots were visualized using enhanced chemiluminescence substrate (Meilunbio, Shanghai, China). The gray value of the target protein band on the membrane was calculated using Image *J* software (NIH, Bethesda, MD, RRID: SCR_003070).

### Gelatin zymography assay

The protein concentration was measured using a BCA kit (Meilunbio, Shanghai, China). The extracted protein (60 mg) with 4 × Zymography Sample Buffer in a volume ratio of 3:1 and then loaded onto 10% sodium dodecyl sulfate-polyacrylamide gels mixed with 15% gelatin for electrophoresis. SDS was removed from the gels to renature the protein with 2.5% Triton X-100 three times for 20 min each. Incubated the gels in 1 × LSCB buffer (10 ×: Ph = 7.6, 0.5 M Tris Base, 2.0 M NaCl, 0.05 M CaCl2, 0.2% (w/v) Brij-35) for 72 h at 37 °C. The gels were incubated with Working Coomassie-G Dye (12.5% Coomassie Brilliant Blue, 10% acetic acid, 50% methanol, 27.5% ddH2O) at 80 rpm for 1 h and washed with decoloring solution (10% acetic acid, 20% methanol, 70% ddH2O). Results were recorded using a scanner (Brother, Shanghai, China).

**Table 1:** RT-qPCR primer sequences.

Gene name	Forward primer (5′-3′)	Reverse primer (5′-3′)
MMP9	ATGTCACTTTCCCTTCACCT	TTAGAGCCACGACCATACAG
MMP2	CTGATAACCTGGATGCAGTCGT	CCAGCCAGTCCGATTTGA
IL-10	GCTCTTACTGGCTGGAGTGAG	CTCAGCTCTCGGAGCATGTG
TGFβ	CCACCTCCTGAGGGTGCTT	ATGTCGGAGAAAGGCGCG
bFGF	CAACACTTACCGGTCACGGA	CCCCGTTTTGGATCCGAGTT
IFNγ	GTCATCGAATCGCACCTGA	GTGCTGGATCTGTGGGTTG
IL-6	TCCTACCCCAACTTCCAATGCTC	TTGGATGGTCTTGGTCCTTAGCC
iNOS	TTYGATGTGCTGCCTCTGGT	CATTCTGCTTCTGGAAACTATGG
IL-1β	CACCTCTCAAGCAGAGCACAG	GGGTTCCATGGTGAAGTCAAC
TNFα	AAATGGGCTCCCTCTCATCAGTTC	TCTGCTTGGTGGTTTGCTACGAC
GAPDH	GATGGTGAAGGTCGGTGTGA	TGAACTTGCCGTGGGTAGAG

### Real-time quantitative PCR (RT-qPCR)

The ipsilateral cortex was collected during cryosectioning and isolated using TRIzol reagent (TR118, Mrcgene, Cincinnati, OH, USA) to extract RNA. RNA concentration was measured using a spectrophotometer (NanoDrop1000, Thermo Fisher, Wilmington, DE, USA). The cDNA, which was reverse transcribed from RNA with an RT Kit (Yeasen, Shanghai, China), was used to perform Real-Time PCR using a SYBR Green Kit (Yeasen, Shanghai, China) under the following cycling conditions:95 °C for 5 min followed by 40 cycles of 95 °C for 10 s and 60 °C for 30 s. The primer sequences for the rats are listed in Table 1.

### Statistical analysis

All experimental data are expressed as mean ± SD. The value of N in all experimental groups was the number of rats. The sample size was determined based on the results of our preliminary experiments and the suggestion from the “Powerandsamplesize.com” website. All data sets were tested for normality by IBM SPSS Statistics software. To evaluate the difference between the two independent groups, an unpaired two-tailed t test was used for variables with normal distribution. One-way ANOVA was used for analysis beyond the two groups, and two-way ANOVA was used for two-factor analysis of variance. Use non-parametric analysis for data without normal distribution. GraphPad software was used for the analysis, and *p*<0.05 was regarded as statistically significant.

## RESULTS

### Optogenetic activation of astrocytes improved neurobehavioral outcomes in the acute phase of ischemic stroke

**Figure 1.**
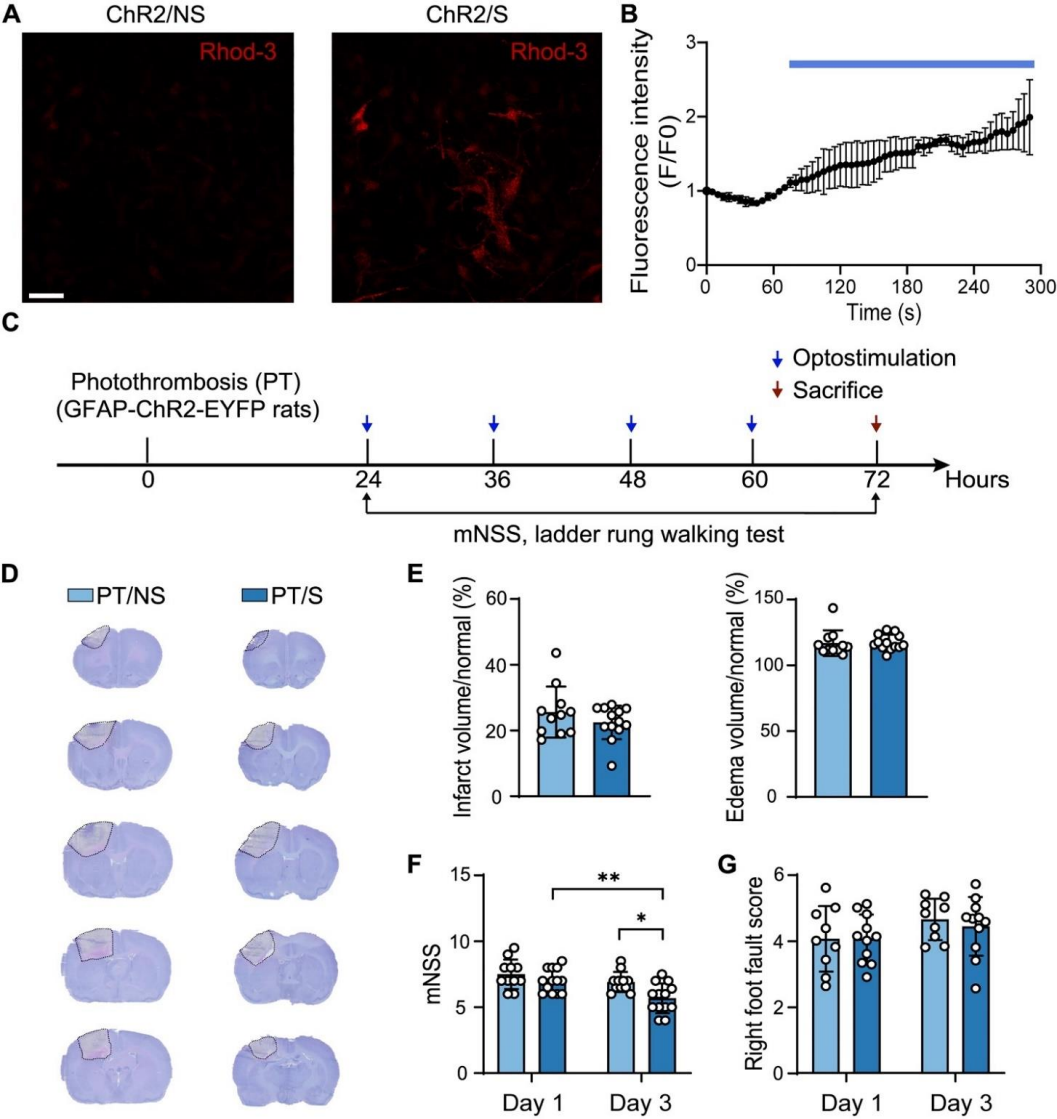
Optogenetic activation of astrocytes improved neurobehavioral outcomes of rats in the acute phase of ischemic stroke. (A) The fluorescence images of Rhod-3 in astrocytes before and after photogenetic stimulation. Scale bar = 50-µm. (B) The statistical results of Rhod-3 fluorescence intensity in astrocytes before and after laser stimulation. F0 is the basic fluorescence of the first ten seconds. (N = 3 fields/group). (C) Animal experiment design *in vivo*. The blue arrow represents opto-stimulation, stimulate for 15 min each time. The red arrow represents sacrificial rats. (D) Representative images of cresyl violet staining at day 3 after PT stroke. Dashed lines indicate cerebral infarction. (E) Quantification the percentage of brain infarct volume (left bar graph) and edema volume (right bar graph) at day 3 after stroke. (N = 11 rats in PT/NS group. N = 13 rats in PT/S group). (F) mNSS at day 1 and day 3 after stroke. (N = 11 rats in PT/NS group. N = 13 rats in PT/S group). Two-way ANOVA test. *p<0.05, **p<0.01. (G) The statistic results of ladder rung walking test at day 1 and day 3 after stroke. (N = 9 rats in PT/NS group. N = 11 rats in PT/S group).

To confirm whether astrocytes could be activated by external laser stimulation, we cultured primary cortical astrocytes form transgenic rats and incubated them with Rhod-3 calcium dye. Calcium levels significantly increased after blue pulse laser stimulation (Supplementary Video. 1), indicating that optostimulation successfully activated astrocytes (Fig. 1A and 1B). The experimental design is illustrated in Fig. 1C.

To investigate the role of optogenetic activation of astrocytes in brain tissue repair in PT stroke rats, we stained brain slices using cresyl violet to measure the volume of cerebral infarct and edema at day 3 after PT stroke (Fig. 1D) and found that the volume of cerebral infarct and edema had a decreasing trend, but there was no difference in the photothrombotic with laser stimulation (PT/S) group compared to the photothrombotic without laser stimulation (PT/NS) group (Fig. 1E). However, the mNSS for neurological function significantly decreased in the PT/S group compared to the PT/NS group on day 3, while no difference was observed on day 1 after PT stroke. There was no difference in the ladder walking test for motor function detection on days 1 and 3 after the PT stroke (Fig. 1F).

**Figure 2.**
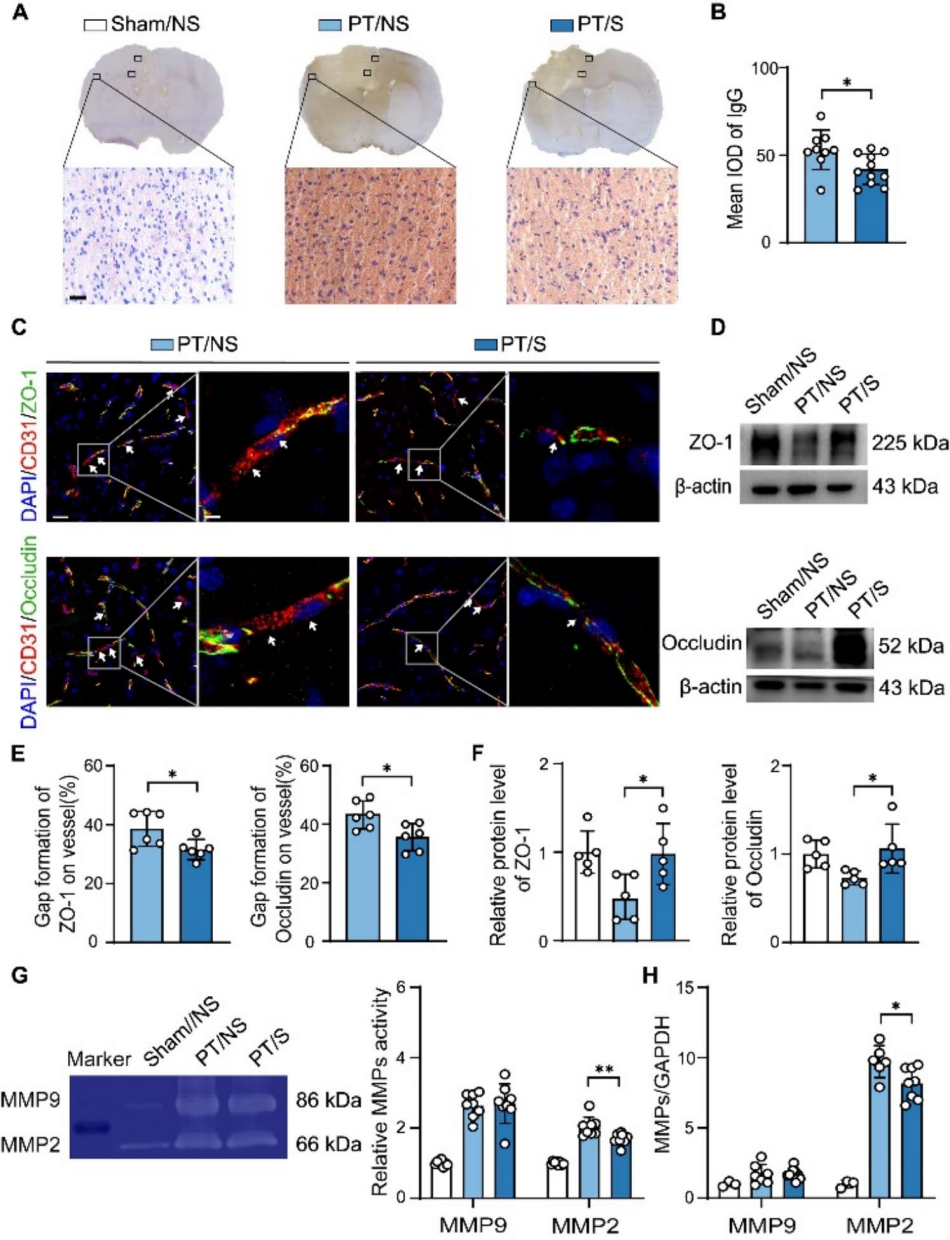
Optogenetic activation of astrocytes attenuated BBB damage in the acute phase of ischemic stroke. (A) Representative images of IgG staining in the Sham/NS, PT/NS and PT/S groups at day 3 in PT stroke rats. Scale bar = 50-µm. The box was the statistical area. (B) Semi-quantitative analysis for the leakage of IgG protein in PT/NS and PT/S groups. (N = 9 rats in PT/NS group. N = 11 rats in PT/S group). Two-tailed t test. *p<0.05. (C) Representative images of tight junction protein ZO-1 (green) or Occludin (green) with endothelial marker CD31 (red) in the peri-infarct region of brain. The arrow points to the gaps in the microvascular after stroke. Left scale bar = 25-µm, right scale bar = 5-µm. (D) Western blot detected the expression of ZO-1 and Occludin. (E) Statistical results of gaps formation in microvascular for ZO-1 (left bar graph) and Occludin (right bar graph). (N = 6 rats/group). Two-tailed t test. *p<0.05. (F) Quantifying ZO-1 (left bar graph) and Occludin (right bar graph) relative to β-actin and normalized to sham/NS group. (N = 5 rats/group). One-way ANOVA test. *p<0.05. (G) Gelatin zymogram (left image) of MMP2 and MMP9 protein activity and quantification (right bar graph) normalized to sham/NS group. (N = 8 rats/group). One-way ANOVA test. **p<0.01. (H) RT-qPCR detected the expression of MMP9 and MMP2 relative to GAPDH and normalized to sham/NS group. (N = 3 rats in Sham/NS group. N = 6-7 rats in PT/NS group. N = 8 rats in PT/S group). One-way ANOVA test. *p<0.05.

### Optogenetic activation of astrocytes attenuated BBB damage in the acute phase of ischemic stroke

Destruction of the BBB is the main pathological feature of the acute phase of ischemic stroke [29]. We detected BBB leakage by IgG staining on day 3 after PT stroke to determine whether the optogenetic activation of astrocytes affects BBB. The results showed that IgG extravasation was alleviated in the PT/S group compared to the PT/NS group (Fig. 2A), suggesting that astrocytes activated by optogenetics could attenuate BBB leakage (Fig. 2B). Furthermore, CD31/ZO-1 and CD31/Occludin fluorescent staining results showed that the disconnection of the vasculature decreased in the PT/S group compared to the PT/NS group (Fig. 2C and 2E). Simultaneously, western blotting results showed that the levels of tight junction proteins increased after laser stimulation (Fig. 2D and 2F). Then, we further examined the activity and expression of matrix metallopeptidase 2/matrix metallopeptidase 9 (MMP2/MMP9), which were related to the BBB integrity. The results showed that MMP2 activity and expression decreased in the PT/S group compared to the PT/NS group, while there was no change in the MMP9 activity and expression (Fig. 2G and 2H). To further explored which cell type release MMPs under the influence of opto-stimulation. We examined the expression of MMP2 and MMP9 in astrocytes [30] and neurons [31, 32] in the PT/NS and PT/S groups and found that optostimulation can reduce the release of MMP2 from astrocytes and neurons (Supplementary Fig. 3). These results suggest that optogenetically activated astrocytes can improve BBB integrity by decreasing the activity and expression of MMP2 in the acute phase of PT stroke in rats.

### IL-10 upregulated in astrocytes after optogenetic activation

**Figure 3.**
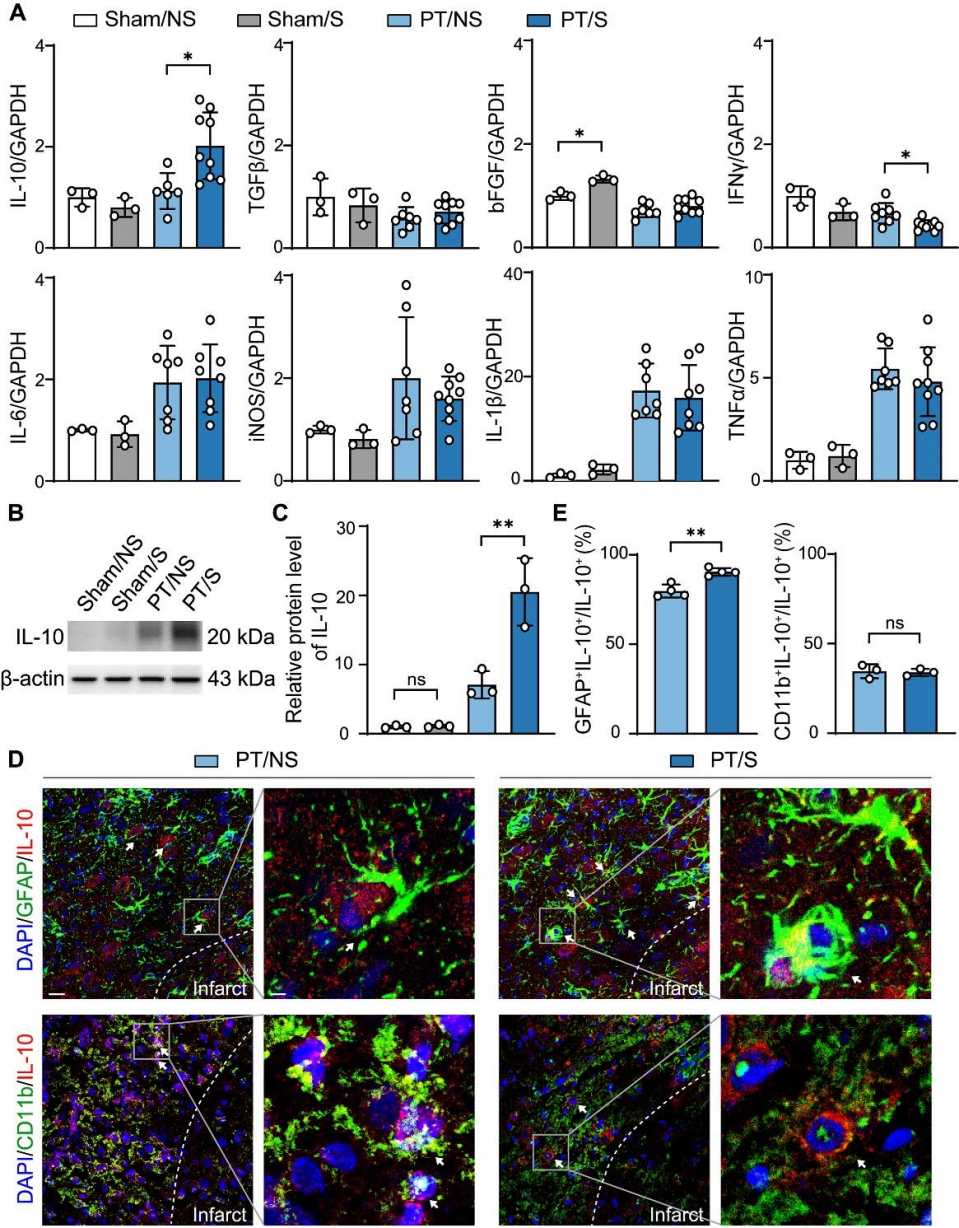
The upregulation of IL-10 in astrocytes after optogenetic activation. (A) RT-qPCR detected the expression of anti-inflammatory factor IL-10, TGF-β and bFGF and pro-inflammatory factors IFNγ, IL-1β, TNFα, IL-6 and iNOS in the sham/NS, sham/S, PT/NS and PT/S groups relative to GAPDH and normalized to sham/NS group at day 3 after PT stroke. (N = 3 rats in Sham/NS group. N = 3 rats in Sham/S group. N = 6-8 rats in PT/NS group. N = 8-9 rats in PT/S group). One-way ANOVA test. *p<0.05. (B) Western blot detected the expression of IL-10. (C) Quantification of IL-10 relative to β-actin and normalized to sham/NS group. (N = 3 rats/group). One-way ANOVA test. **p<0.01. (D) Representative immunofluorescent images of GFAP (green) or CD11b (green) and IL-10 (red) in the peri-infarct region. The arrow points to the colocalization cells. Left scale bar = 25-µm, right scale bar = 5-µm. (E) Quantitative analysis of IL-10 expression in astrocytes and microglia: the percentage of GFAP^+^ IL-10^+^ (N = 4 rats/group) or CD11b^+^ IL-10^+^ (N = 3 rats/group). cell in the total IL-10^+^ cells. Two-tailed t test. **p<0.01.

To explore the protective mechanism of the optogenetic activation of astrocytes on the BBB, we further examined the expression of inflammatory factors in the brain after PT stroke. The results showed that the expression of IL-10 increased in the PT/S group compared to the sham without laser stimulation (Sham/NS), sham with laser stimulation (Sham/S), and PT/NS groups, while the expression of TGF-β and bFGF did not change. Correspondingly, IFNγ decreased after optogenetic activation, whereas IL-1β, TNFα, IL-6 and iNOS expression did not differ among the groups (Fig. 3A). These results indicated that the protective effect of optogenetically activated astrocytes in the acute phase of stroke was exerted by increasing the IL-10 to regulate the inflammatory environment. Furthermore, western blot analysis showed that the expression of IL-10 increased after optogenetic activation (Fig. 3B). IL-10 expression was not different between the Sham/NS and Sham/S groups of rats (Fig. 3C), indicating that photogenetic-activated astrocytes caused the upregulation of IL-10 after PT stroke, but it was not affected under normal conditions.

Since both astrocytes and microglia/macrophages can release IL-10 [33, 34], we used double immunostaining of GFAP/IL-10 and CD11b/IL-10 to explore which cell type contributed to the increased IL-10 after optogenetic activation in PT stroke rats. The results showed that IL-10 expression increased in astrocytes, while there was no difference in microglia/macrophages between PT/S and PT/NS rats (Fig. 3D-E). These results indicate that the increased IL-10 in PT stroke rats after optogenetic activation was derived from astrocytes, but not microglia/macrophages.

### The increase of IL-10 in astrocytes reduced the neuronal apoptosis in the acute stage in PT stroke rats

**Figure 4.**
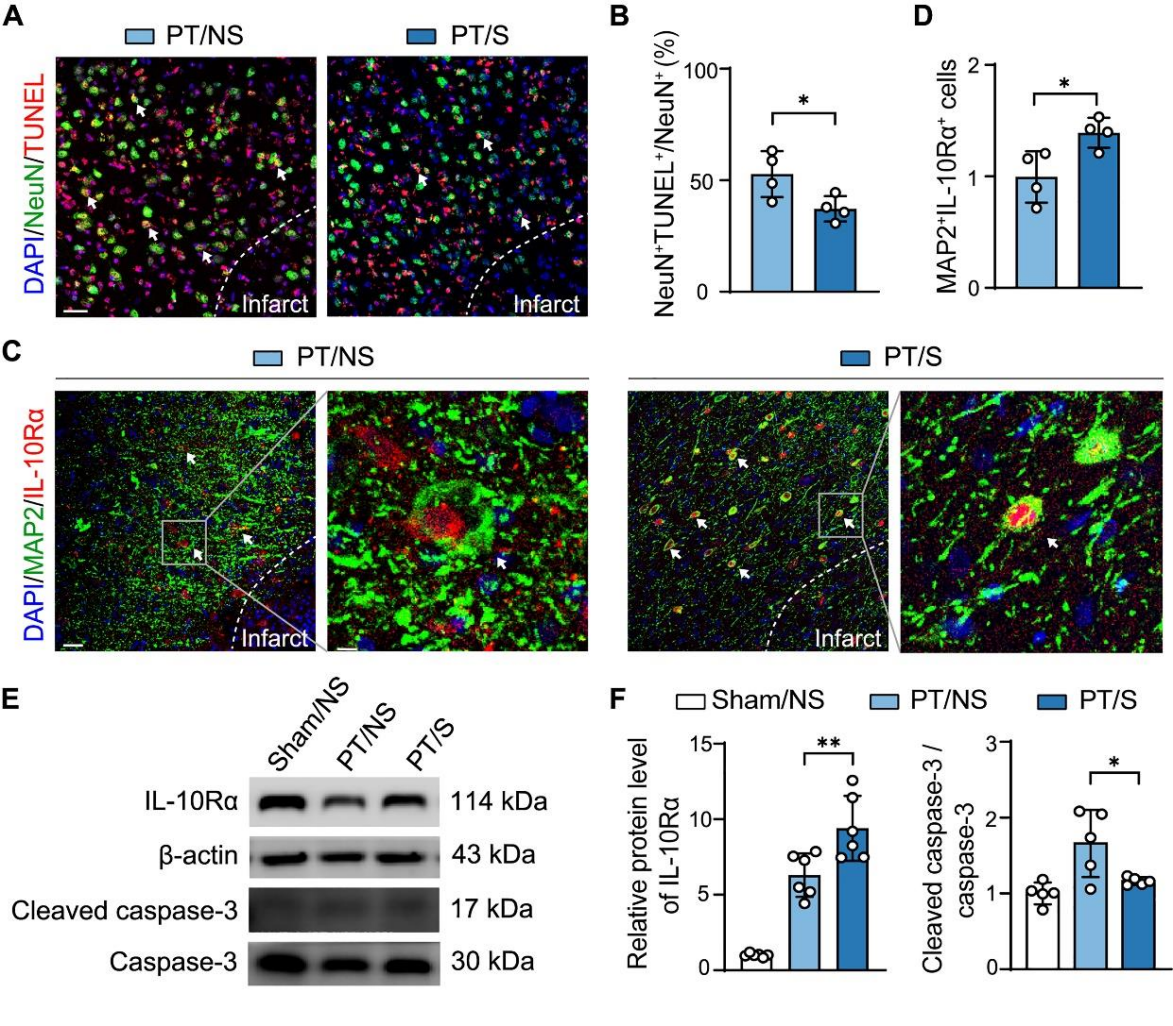
The increase of IL-10 in astrocytes further reduced the neuronal apoptosis in the acute stage of ischemic stroke. (A) Representative TUNEL (red) and NeuN (green) immunostaining images in the peri-infarct region in the PT/NS and PT/S groups at day 3 after stroke. The arrow points to the colocalization cells. Scale bar = 50-µm. (B) Statistical results of neuronal apoptosis: the percentage of TUNEL^+^ NeuN^+^ cell number in the total number of NeuN^+^ cells. (N = 4 rats/group). Two-tailed t test. *p<0.05. (C) Representative immunofluorescent images of MAP2 (green) and IL-10Rα (red) in the peri-infarct region. The arrow points to the colocalization cells. Left scale bar = 25-µm, right scale bar = 5-µm. (D) Quantitative analysis of IL-10Rα expression in neurons: the number of MAP2^+^ IL-10Rα^+^ cells normalized to PT/NS group. (N = 4 rats/group). Two-tailed t test. *p<0.05. (E) Western blot detected the expression of IL-10Rα, Cleaved caspase-3 and Caspase-3. (F) Quantifying IL-10Rα (N = 6 rats/group, left bar graph) and cleaved caspase-3 and caspase-3 (N = 5 rats/group, right bar graph) expression and normalized to sham/NS group in ipsilateral hemisphere of rat brain after stroke. One-way ANOVA test. *p<0.05, **p<0.01.

Since IL-10 released from astrocytes has a protective effect on neurons [35], we detected neuronal apoptosis by TUNEL staining after optogenetic activation. The results showed that neuronal apoptosis was reduced in the peri-infarct area of PT/S rats compared with that in PT/NS rats (Fig. 4A and 4B). To further examine whether the protective effect of the optogenetic activation of astrocytes on cortical neurons is due to IL-10, we examined IL-10Rα in neurons to determine whether IL-10 derived from astrocytes could decrease neuronal apoptosis via IL-10Rα. The results showed that Neuronal IL-10Rα expression was higher in the PT/S group than in the PT/NS group (Fig. 4C and 4D). Simultaneously, we detected that cleaved caspase-3, caspase-3 and IL-10Rα protein expression in the Sham/NS, PT/NS, and PT/S groups. The results showed that cleaved caspase-3/caspase-3 expression decreased and IL-10Rα expression increased in the PT/S group compared to those in the PT/NS group (Fig. 4E and 4F). These results indicate that IL-10 released by the optogenetic activation of astrocytes may act on IL-10 receptors in neurons to reduce neuronal apoptosis.

### Inhibiting IL-10 in astrocytes aggravated BBB damage after PT stroke

To further verify that the protective effect of optogenetically activated astrocytes on the BBB is due to IL-10, we inhibited the expression of IL-10 in astrocytes using HBAAV2/9-GFAP-mir30-r-IL-10-mCherry. HBAAV2/9-GFAP-mcherry was used as a scrambled control (Supplementary Fig. 4). The interference efficiency of the virus in the peri-infarct area was approximately 80% (Supplementary Fig. 1C).

**Figure 5.**
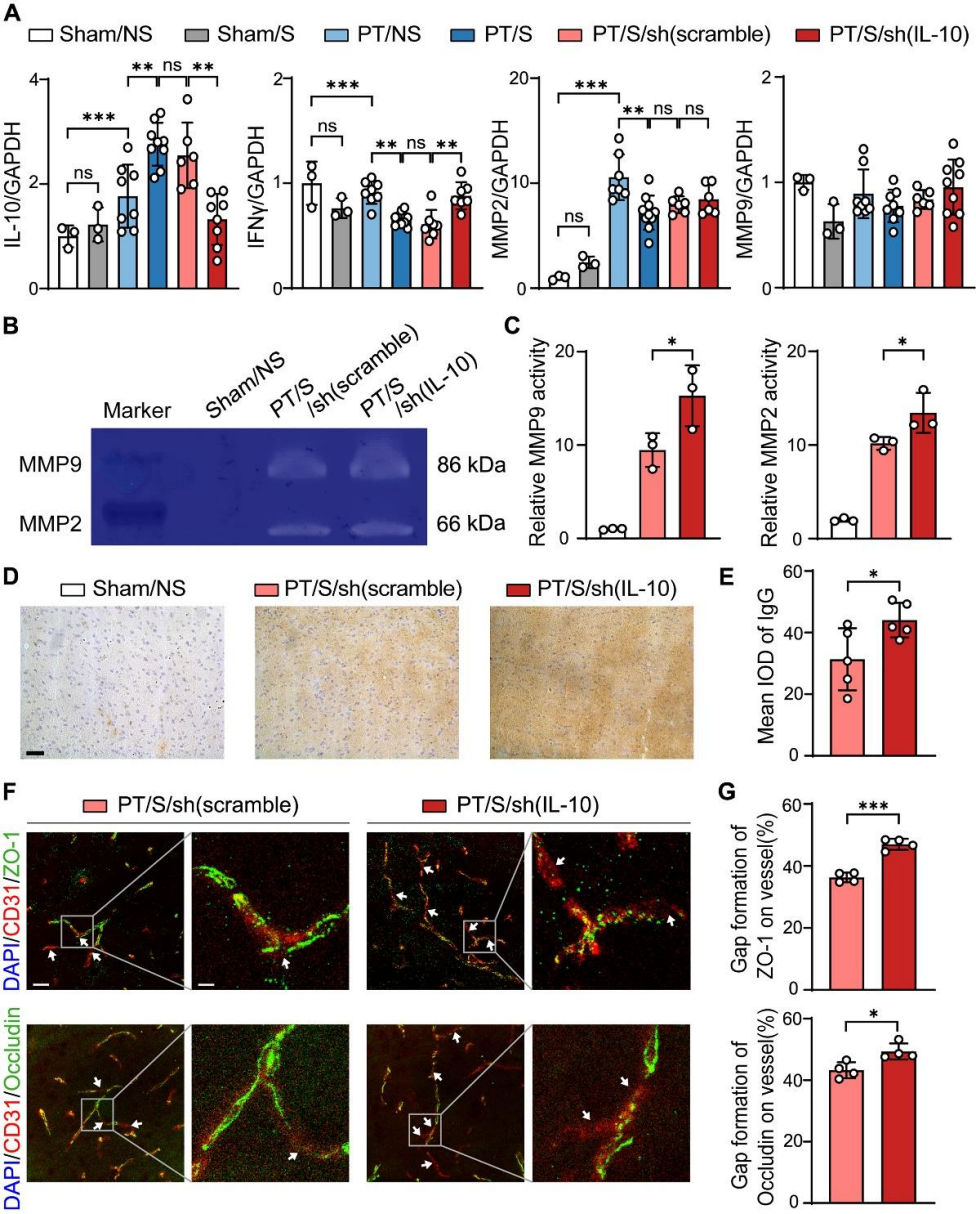
Inhibition of IL-10 in astrocytes aggravated the damage of BBB after ischemic stroke. (A) RT-qPCR detected the expression of IL-10, IFNγ, MMP2 and MMP9 in the sham/NS, sham/S, PT/NS, PT/S, PT/S/sh (scramble) and PT/S/sh (IL-10) groups relative to GAPDH and normalized to sham/NS group at day 3 after PT stroke. (N = 3 rats in Sham/NS group. N = 3 rats in Sham/S group. N = 7-8 rats in PT/NS group. N = 8-10 rats in PT/S group. N = 6-7 rats in PT/S/sh (scramble) group. N = 6-9 rats in PT/S/sh (IL-10) group). One-way ANOVA test. *p<0.05, **p<0.01, ***p<0.001. (B) Gelatin zymogram detected the activity of MMP9 and MMP2 protein in the sham/NS, PT/S/sh (scramble) and PT/S/sh (IL-10) groups of rats at day 3 after PT stroke. (C) Quantification of MMP9 (left bar graph) and MMP2 (right bar graph) protein activity normalized to sham/NS group. (N = 3 rats/group). One-way ANOVA test. *p<0.05. (D) Representative images of IgG staining in the sham/NS, PT/S/sh (scramble) and PT/S/sh (IL-10) groups at the day 3 after PT stroke. Scale bar = 50-µm. (E) Semi-quantitative analysis for the leakage of IgG protein. (N = 5 rats/group). Two-tailed t test. *p<0.05. (F) Representative immunofluorescent images of tight junction protein ZO-1 (green) or Occludin (green) and endothelial marker CD31 (red) in the peri-infarct region. The arrow points to the colocalization cells. Left scale bar = 25-µm, right scale bar = 5-µm. (G) Statistical results for gap formation in microvascular of ZO-1 and Occludin in the peri-infarct region. (N = 4 rats/group). Two-tailed t test. *p<0.05. ***p<0.001.

We used RT-qPCR to detect the expression of inflammatory factors in the brain. The results showed that the expression of IL-10 increased after stroke, and this upregulation was more significant in the PT/S group than in the PT/NS group. This increase was inhibited in the photothrombotic with laser stimulation and sh (IL-10) AAV (PT/S/sh (IL-10)) groups (Fig. 5A), which indicated that the inhibition of IL-10 in astrocytes was successful. At the same time, the expression of IFNγ in the PT/S/sh (IL-10) group was higher than that in the photothrombotic with laser stimulation and sh (scramble) AAV (PT/S/sh (scramble)) groups (Fig. 5A), which suggested that virus interference could reverse regulating optogenetic stimulation by decreasing the release of anti-inflammatory factors.

**Figure 6.**
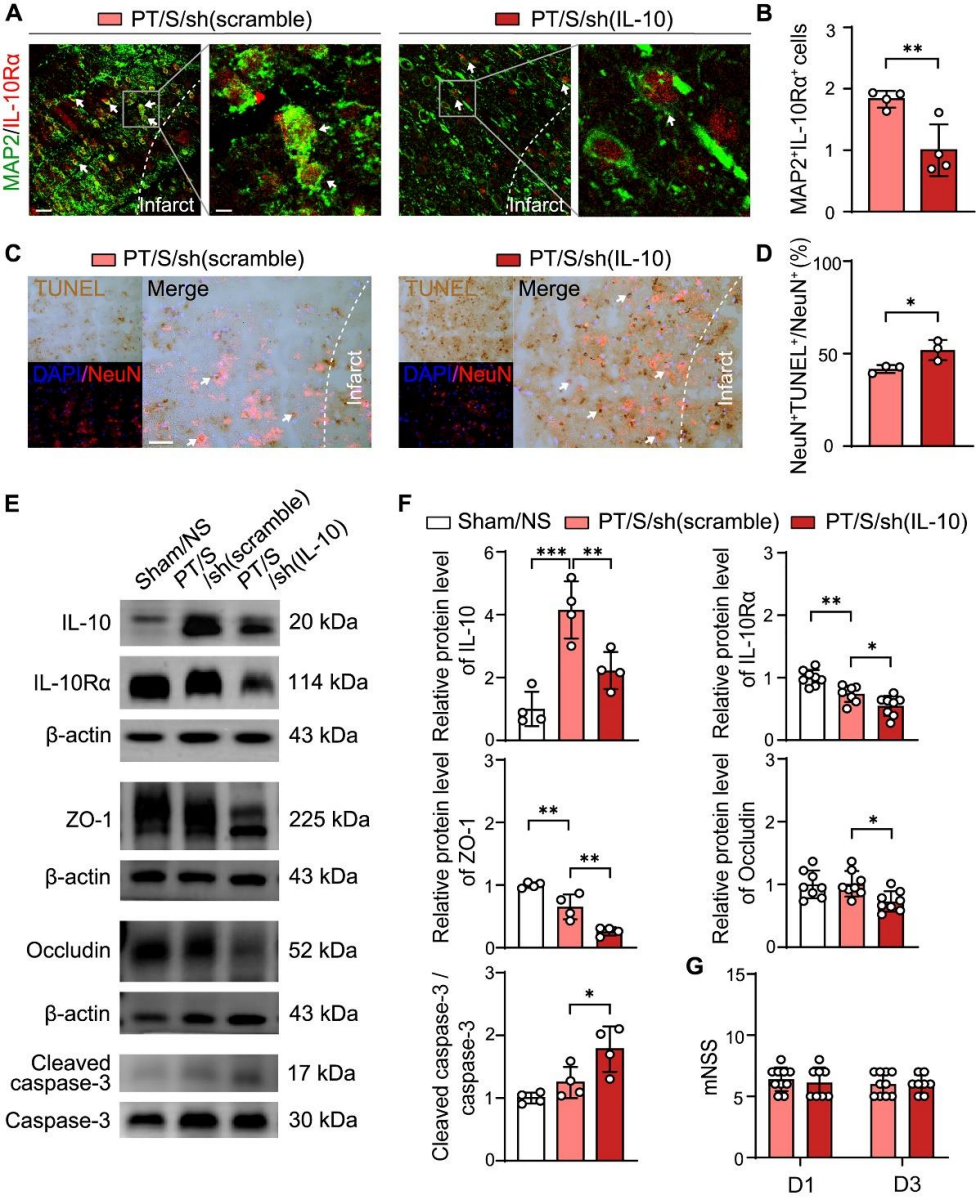
Astrocytic IL-10 inhibition increased the apoptosis of neurons. (A) Representative immunofluorescent images of MAP2 (green) and IL-10Rα (red) in the peri-infarct region in PT/S/sh (scramble) and PT/S/sh (IL-10) groups at day 3 after stroke. The arrow points to the colocalized cells. Left scale bar = 25-µm, right scale bar = 5-µm. (B) Quantitative analysis for the expression of IL-10Rα in neurons: the number of MAP2^+^ IL-10R^+^ cells normalized to PT/S/sh (IL-10) group. (N = 4 rats/group). Two-tailed t test. **p<0.01. (C) The representative immunofluorescent images of TUNEL (brown, chromogenic method) and NeuN (red) immunostaining in the peri-infarct region. Left scale bar = 50-µm, right scale bar = 10-µm. (D) Statistical results for the percentage of TUNEL^+^ NeuN^+^ cell in the total NeuN+ cells. (N = 3 rats/group). Two-tailed t test. *p<0.05. (E) Western blot detected the expression of IL-10, IL-10Rα, ZO-1, Occludin, Cleaved caspase-3 and Caspase-3 in Sham/NS, PT/S/sh (scramble) and PT/S/sh (IL-10) groups. (F) Quantification of protein level of IL-10, IL-10Rα, ZO-1, Occludin, Cleaved caspase-3 and Caspase-3 expression and normalized to sham/NS group at day 3 after PT stroke. (N = 4-8 rats in Sham/NS group. N = 4-8 rats in PT/S/sh (scramble) group. N = 4-8 rats in PT/S/sh (IL-10) group). One-way ANOVA test. *p<0.05, **p<0.01, ***p<0.001. (G) Statistical results of mNSS at day 1 and day 3 in PT/S/sh (scramble) and PT/S/sh (IL-10) groups. (N = 10 rats in PT/S/sh (scramble) group. N = 8 rats in PT/S/sh (IL-10) group).

To investigate the effect of inhibiting IL-10 expression on BBB integrity after PT stroke, we detected changes in MMP2 and MMP9 by RT-qPCR (Fig. 5A) and their activity by gelatin zymography (Fig. 5B). The results showed that although the overall levels of MMP2 and MMP9 did not change after inhibition, the activity of MMP2 and MMP9 increased in the PT/S/sh (IL-10) group compared to the PT/S/sh (scramble) group (Fig. 5C), indicating that the inflammatory environment of the BBB was more severe after the inhibition of IL-10 in astrocytes. Furthermore, IgG staining showed that BBB leakage increased in the PT/S/sh (IL-10) group compared to that in the PT/S/sh (scramble) group (Fig. 5D and 5E). Immunofluorescence analysis of tight junction proteins showed that the disconnection length of ZO-1 and Occludin in the vascular tissue of the PT/S/sh (IL-10) group was greater than that in the PT/S/sh (scramble) group (Fig. 5F and 5G). These results indicate that inhibition of astrocyte IL-10 can reverse the protective effect of optogenetic stimulation on BBB integrity.

### Astrocytic IL-10 inhibition increased the apoptosis of neurons

To determine whether inhibition of IL-10 in astrocytes also affects neuronal apoptosis after stroke, we detected the expression of IL-10Rα in neurons by immuno-fluorescence. The results showed that the expression of IL-10Rα decreased significantly in the PT/S/sh (IL-10) group compared to that in the PT/S/sh (scramble) group (Fig. 6A and 6B). At the same time, we detected neuronal apoptosis in both groups by TUNEL staining and found that the neuronal apoptosis increased in the inhibiting group (Fig. 6C and D). These results suggest that inhibiting astrocyte release IL-10 could reverse the effects of optogenetic stimulation on neurons after PT stroke.

Western blotting further showed that the expression of IL-10, IL-10Rα, tight junction proteins ZO-1 and Occludin decreased, and cleaved caspase-3/caspase-3 expression increased in the PT/S/sh (IL-10) group compared to the PT/S/sh (scramble) group (Fig. 6E and F). However, there was no difference between the two groups in the neurobehavioral score on day 3 in the PT stroke rats (Fig. 6G). These results indicated that inhibiting IL-10 in astrocytes could reverse the protective effect of optogenetic stimulation on the BBB and neurons.

**Figure 7.**
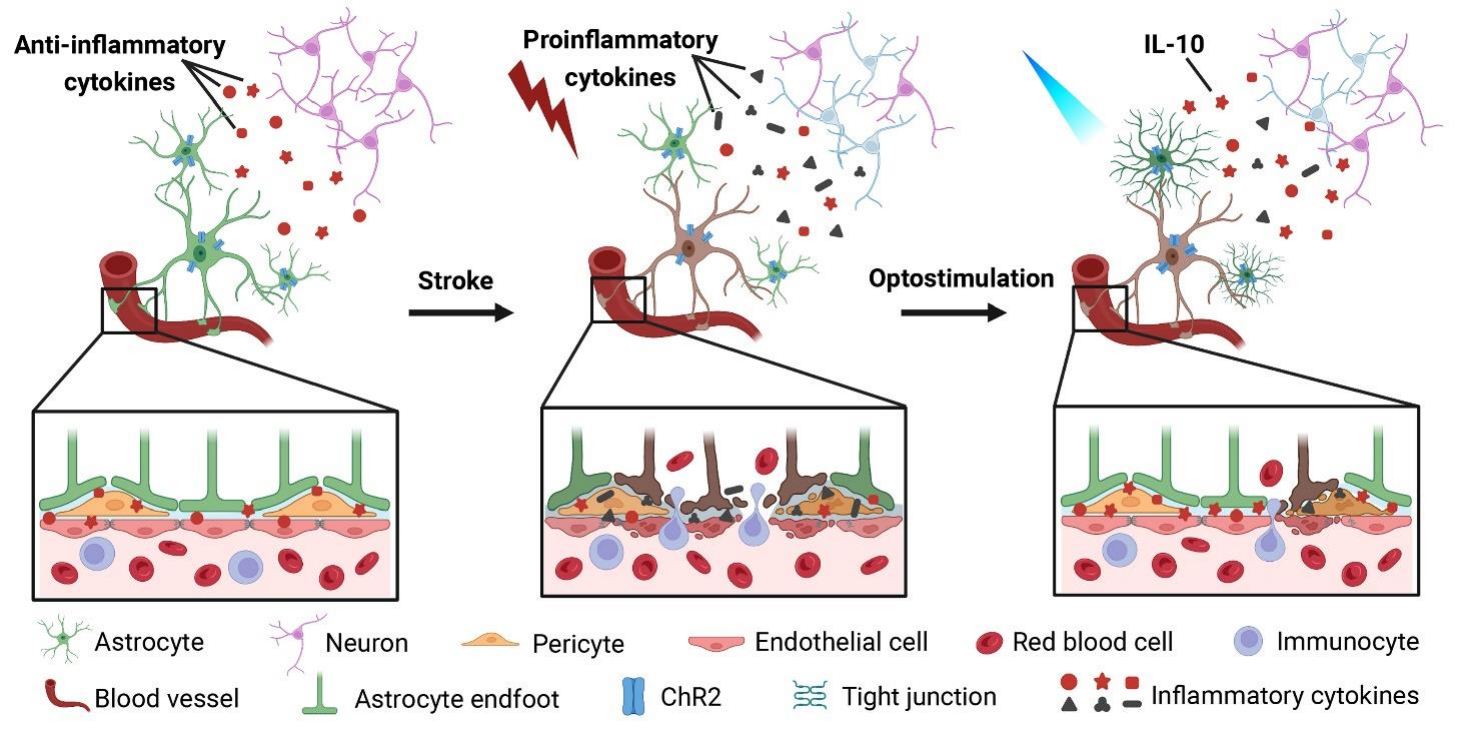
Cartoon diagram of optogenetics-activated astrocytes reducing BBB disruption in ischemic stroke.

## DISCUSSION

Accumulating evidence shows that astrocytes modulate neuronal activity [36, 37], releasing glutamate and ATP for use as gliotransmitters under physiological and pathological condition [38-40]. However, astrocytes are also cardinal components of the BBB that regulate microvessel function. The effects and mechanisms of astrocyte activity on the BBB after stroke remain to be elucidated. Our results showed that astrocyte activation induced by ChR2 optogenetics reduced BBB damage by releasing IL-10, which decreased MMP expression and neuronal apoptosis in PT-stroke rats. First, astrocyte activation alleviates BBB damage and improves functional neurological recovery. Second, astrocyte activation increases IL-10 release and decreases IFNγ and MMP2 expression. Third, the inhibition of astrocytic IL-10 expression aggravates BBB damage, accompanied by an increase in MMP2 and MMP9 activity.

BBB breakage is a cardinal pathological feature of PT stroke. Improving BBB integrity is a therapeutic target for the acute stage of PT stroke. Astrocytes are involved in the regulation of BBB integrity by releasing trophic factors after brain injury [41, 42]. Photo-stimulation of ChR2-expressing astrocytes evokes a rapid and robust increase in cerebral blood flow by K+ channels under physiological conditions, while the effect of photo-stimulated astrocyte on the BBB and its underlying mechanism are elusive [43]. Although ChR2-photoactivated glial cells can increase glutamate and neuronal excitotoxicity after ischemic brain damage [39], our results support the notion that ChR2-photostimulated astrocytes increase tight junction protein ZO-1, Occludin expression and decrease IgG exudation in PT stroke rats. The difference was probably because the first-time photo-stimulation we applied was 24 h after PT stroke, which extends the time window for the clinical stroke therapy, while Kaoru Beppu et al. applied the first stimulation at 3 h after PT [39].

IL-10 is an anti-inflammatory factor that plays a pivotal protective role under many inflammatory conditions. Cerebral ischemic injury is often accompanied by inflammatory responses characterized by the production of many inflammatory factors, which aggravate BBB disruption. IL-10 can inhibit pro-inflammatory cytokines produced by activated glia [44]. Astrocytes are the main source of IL-10 in the central nervous system [45]. Numerous studies have shown that astrocyte-derived IL-10 decreases inflammation and attenuates neuronal death [34, 35, 45, 46]. Astrocyte-targeted IL-10 overproduction reduces neurodegeneration in cortical injury [47]. Endogenous IL-10 can bind to the IL-10 receptor on sensory neurons to regulate their activity, thereby inhibiting neuroinflammation and relieving neuropathic pain [48]. Preconditioning astrocytes to mild oxidative stress increases neural cell survival in an IL-10-dependent manner [49]. IL-10 overexpression is associated with a striking resistance to cerebral ischemia [50, 51]. IL-10 deficiency increases the expression of TNF-α, IL-1b, IL-6, TLR-4 and other pro-inflammatory factors [18, 52]. IL-10 gene transfer with an adenovirus vector reduced infarct volume in a rat model of photothrombotic ischemia [53]. Mice lacking IL-10 show a larger infarction after permanent middle cerebral artery occlusion (MCAO) [54]. In our study, the infarct volume and edema areas did not change significantly after opto-stimulation. The reason could be that only the astrocytes around the laser fiber were affected, which is not enough to change the tissue-level damage. On the other hand, although IL-10 can affect the inflammatory reaction in the brain, it has little effect on the regression of acute lesions [55]. The neurobehavioral outcomes did not improve after IL-10 expression inhibition, which could also be due to the complex crosstalk regulation and immune compensation mechanism between resident glial cells and infiltrated immune cells [56].

MMPs exacerbates secondary inflammation at day 4 after MCAO [55], and IL-10 can modulate MMPs expression during the development of bronchopulmonary dysplasia [57, 58]. Perivascular astrocytes release cytokines and activate MMPs, which contribute to BBB disruption and vasogenic edema after ischemic stroke [59]. Our data support that optogenetically activated astrocytes protect BBB integrity by decreasing IFNγ expression and MMP2 activity and inhibiting neuronal apoptosis by IL-10. Since IL-10 receptor (IL-10R) has been found to be expressed on microglia [60, 61], astrocytes [62, 63], oligodendrocytes [64], and neurons [63, 65], we speculated that astrocytic IL-10 released from optogenetics-activated astrocytes acts on itself receptor to regulate MMPs activity and neuron survival. Our data showed that ChR2 stimulation decreased MMP2 activity, while GFAP-IL-10sh increased MMPs activity. We also found that the activities of MMP2 and MMP9 increased after treatment with GFAP-IL-10sh. Simultaneously, we observed that optogenetic activation of astrocytes can reduce the release of MMP2 from neurons, which is consistent with the phenomenon that MMPs activity can promote neuronal apoptosis in acute stroke injury [32]. Therefore, we speculate that the optogenetic activation of astrocytes releases IL-10, which acts on neurons through IL-10R, reducing the expression of MMPs in neurons to reduce neuronal apoptosis. However, the mechanism by which IL-10 derived from astrocytes regulates MMPs expression and activity needs to be explored in the future.

## Conclusions

Our study demonstrated that optogenetically activated astrocytes protect BBB integrity and reduce neuronal apoptosis by upregulating IL-10, which decreases MMPs activity and ameliorates inflammation (Fig. 7), thus providing a novel approach for stroke treatment.

## Supplementary Materials

The Supplementary data can be found online at: www.aginganddisease.org/EN/10.14336/AD.2023.0226.


