# Supplementary material for: Optogenetic Activation of Astrocytes Reduces Blood-Brain Barrier Disruption via IL-10 In Stroke
Source: Aging Dis. 2023 Oct 1;14(5):1870–86. doi: 10.14336/AD.2023.0226 (PMC10529757; doi:10.14336/AD.2023.0226)
Supplement: Supplementary file 1 [file AD-14-5-1870-s.pdf]

SUPPLEMENTARY DATA

Optogenetic Activation of Astrocytes Reduces Blood-Brain Barrier Disruption *via* IL-10 In Stroke

Qian Suo, Lidong Deng, Tingting Chen, Shengju Wu, Lin Qi, Ze Liu, Tingting He, Heng-Li Tian, Wanlu Li, Yaohui Tang, Guo-Yuan Yang, Zhijun Zhang[*]

© 2023. Suo Q et al. Published online at http://www.aginganddisease.org/EN/10.14336/AD.2023.0226

SUPPLEMENTARY DATA

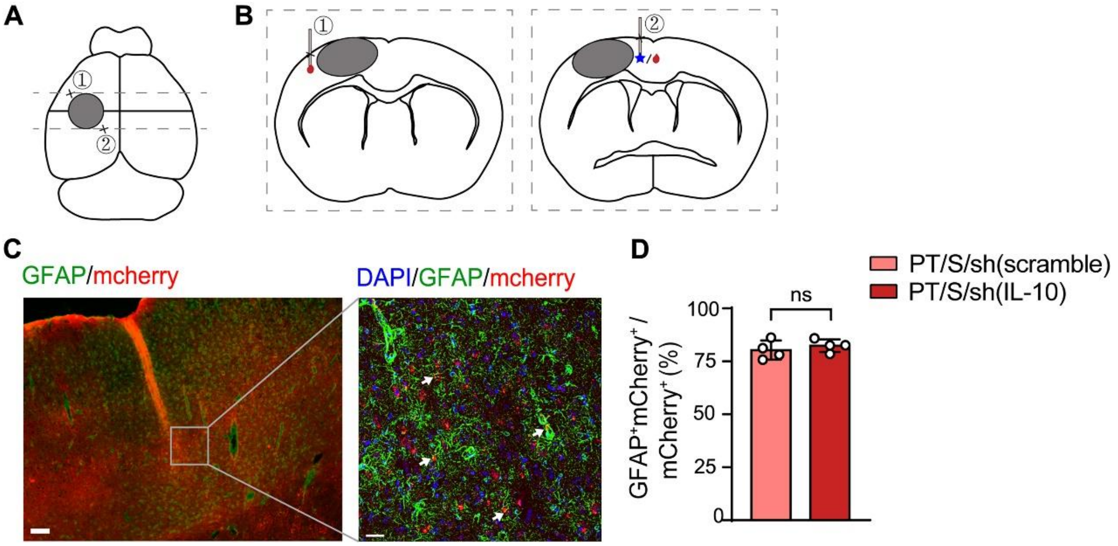

Supplementary Figure 1. Schematic diagram of virus injection site and expression. (**A**) The horizontal section of rat brain and gray area indicates infarction. (B) The left panel shows the coronal section of No.1 site and red area indicates the location of virus injection. The right panel shows the coronal section of No.2 site, the red area indicates the location of virus injection, and the blue area indicates the opto-stimulation. (C) Schematic diagram of virus expression of DAPI (blue), GFAP (green) and mCherry (red) in the peri-infarct region in Sham/NS/sh (scramble) groups. Virus expression statistics is the percentage of GFAP+mCherry+ cell number in the total number of GFAP+ cells. (N = 4 rats/group). Left scale bar = 100 µm, right scale bar = 25 µm.

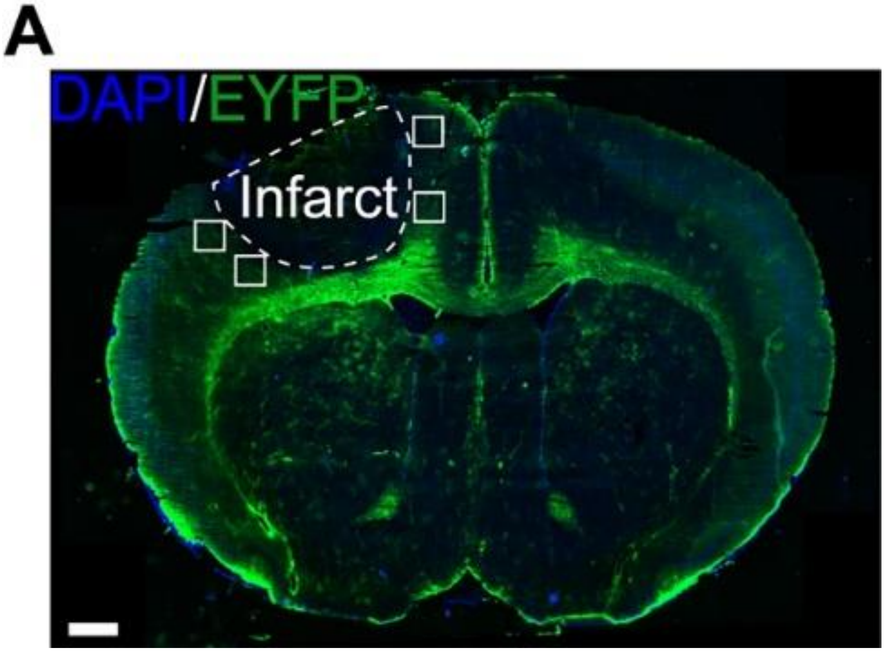

Supplementary Figure 2. Statistical schematic diagram of immunofluorescence staining. (A) Schematic diagram of large picture splicing in PT/S group, DAPI (blue) shows the total brain area, EYFP (green) stands for optogenetics-activated astrocytes, the dotted line area represents the infarction area, and the four rectangular areas are the fluorescence statistical positions. Scale bar = 50 µm.

© 2023. Suo Q et al. Published online at http://www.aginganddisease.org/EN/10.14336/AD.2023.0226

SUPPLEMENTARY DATA

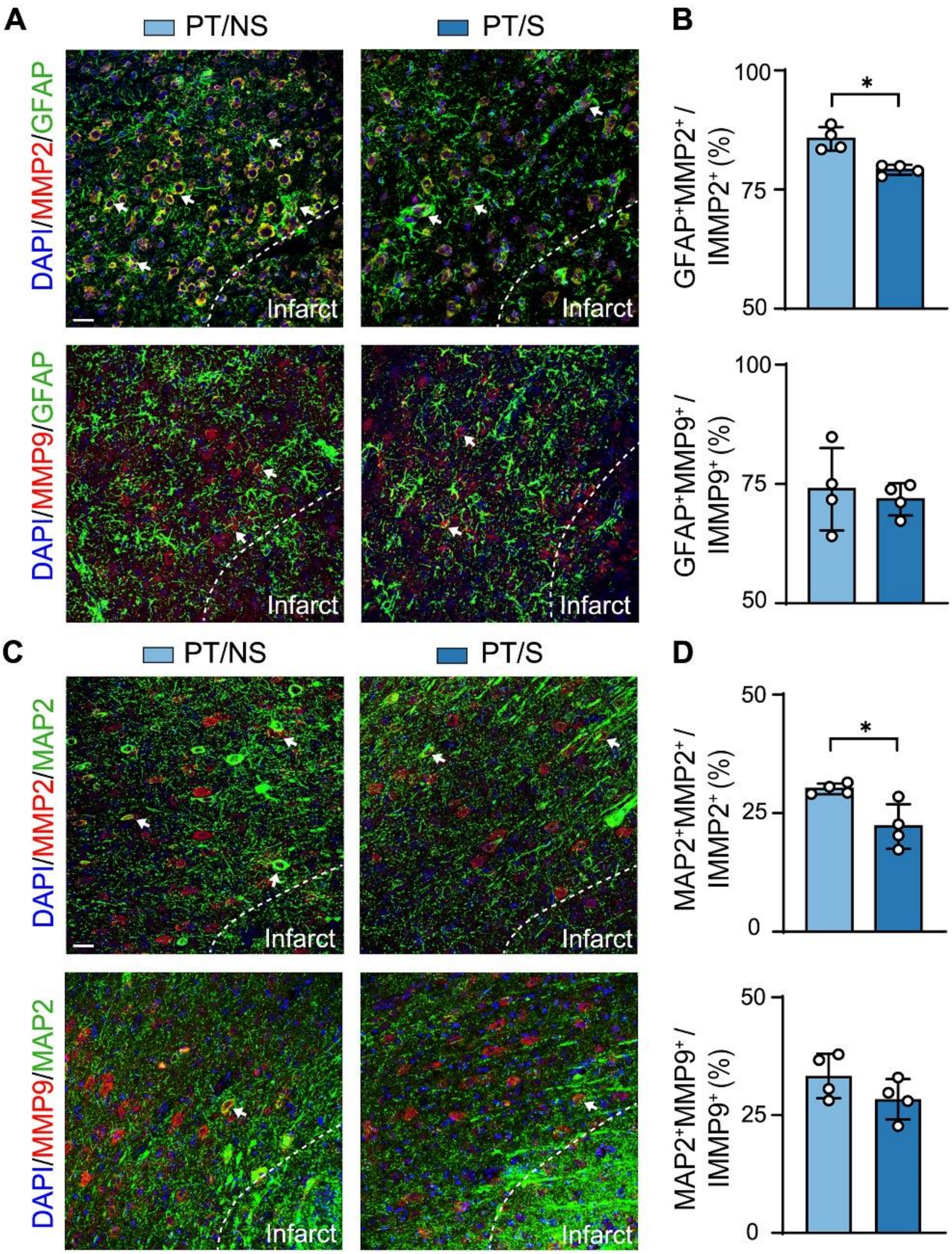

Supplementary Figure 3. Expression of MMP-2 and MMP9 in astrocytes and neurons. (A) Representative immunofluorescent images of DAPI (blue), astrocytes marker GFAP (green) and MMP2/MMP9 (red) in the peri-infarct region. The arrow points to the colocalization cells. scale bar = 25 µm. (B) Statistical results of neuronal apoptosis: the percentage of $GFAP^{+}MMP2^{+}$/ $GFAP^{+}MMP9^{+}$ cells in the total number of $MMP2/MMP9^{+}$ cells. (N = 4 rats/group). Two-tailed t test. *p<0.05. (C) Representative immunofluorescent images of DAPI (blue), neurons marker MAP2 (green) and MMP2/MMP9 (red) in the peri-infarct region. The arrow points to the colocalization cells. Left scale bar = 25 µm, right scale bar = 5 µm. (D) Statistical results of neuronal apoptosis: the percentage of $MAP2^{+}MMP2^{+}$/ $MAP2^{+}MMP9^{+}$ cells in the total number of $MMP2/MMP9^{+}$ cells. (N = 4 rats/group). Two-tailed t test. *p<0.05.

© 2023. Suo Q et al. Published online at http://www.aginganddisease.org/EN/10.14336/AD.2023.0226

SUPPLEMENTARY DATA

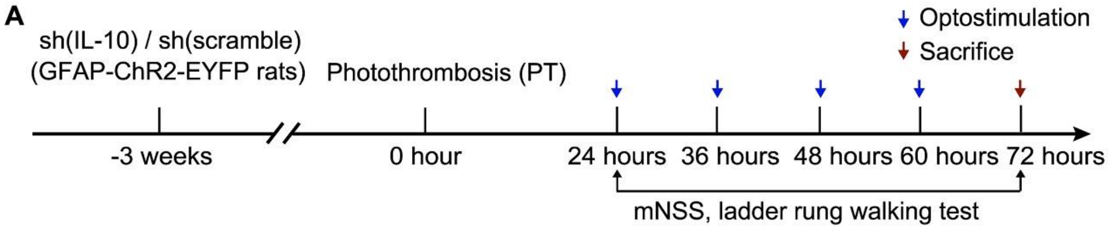

Supplementary Figure 4. Animal experimental design of IL-10 inhibition *in vivo*

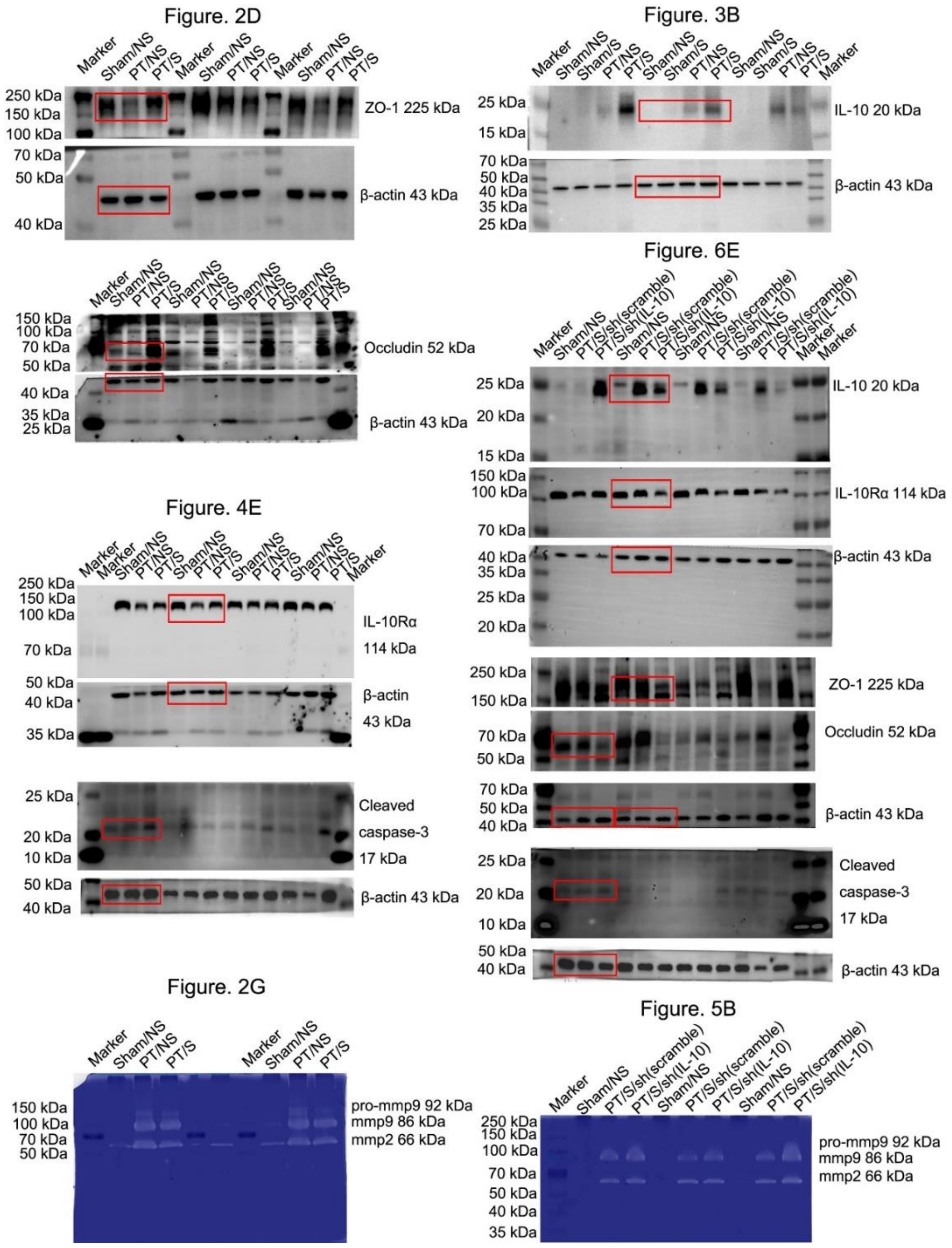

Supplementary Figure 5. Raw data of western blot and gelatin zymogram gel panels.

© 2023. Suo Q et al. Published online at http://www.aginganddisease.org/EN/10.14336/AD.2023.0226

SUPPLEMENTARY DATA

Calcium-rhod3.mp4

Supplementary video 1. Calcium imaging video recording *in vitro*

© 2023. Suo Q et al. Published online at http://www.aginganddisease.org/EN/10.14336/AD.2023.0226
